# Prioritization of arbitrary faces associated to self: An EEG study

**DOI:** 10.1371/journal.pone.0190679

**Published:** 2018-01-02

**Authors:** Mateusz Woźniak, Dimitrios Kourtis, Günther Knoblich

**Affiliations:** 1 Cognition and Philosophy Lab, Department of Philosophy, Monash University, Melbourne, Australia; 2 Institute of Psychology, Jagiellonian University, Cracow, Poland; 3 Department of Cognitive Science, Central European University, Budapest, Hungary; University of Bologna, ITALY

## Abstract

Behavioral and neuroimaging studies have demonstrated that people process preferentially self-related information such as an image of their own face. Furthermore, people rapidly incorporate stimuli into their self-representation even if these stimuli do not have an intrinsic relation to self. In the present study, we investigated the time course of the processes involved in preferential processing of self-related information. In two EEG experiments three unfamiliar faces were identified with verbal labels as either the participant, a friend, or a stranger. Afterwards, participants judged whether two stimuli presented in succession (ISI = 1500ms) matched. In experiment 1, faces were followed by verbal labels and in experiment 2, labels were followed by faces. Both experiments showed the same pattern of behavioral and electrophysiological results. If the first stimulus (face or label) was associated with self, reaction times were faster and the late frontal positivity following the first stimulus was more pronounced. The self-association of the second stimulus (label or face) did not affect response times. However, the central-parietal P3 following presentation of the second stimulus was more pronounced when the second stimulus was preceded by self-related first stimulus. These results indicate that even unfamiliar faces that are associated to self can activate a self-representation. Once the self-representation has been activated the processing of ensuing stimuli is facilitated, irrespective of whether they are associated with the self.

## Introduction

The topic of self has a long history in science and philosophy. However, there is still no general agreement about a definition of self. One important aspect of self that is open to empirical investigation are the attentional mechanisms involved in processing of information related to self as object, or “Me” [1], which denote self as an object of perception or self-attribution [2–4]. This contrasts with views of the self as a subject, or “I”, which refer to the experience of being an agent [1, 5]. Self as object is sometimes further differentiated into subcategories, such as bodily self [6–8], extended self [9], or conceptual self [10, 11], depending on the type of information it comprises. Moreover, self as object can be approached as either a phenomenal structure–the conscious experience of “mineness”, i.e. that something is a part of me or belongs to me (often described as sense of self or a form of self-consciousness, e.g. [7, 8, 12]), or as a mental representation of what is a part of me or belongs to me (for a discussion about the relationship between phenomenal and representational content see, e.g. [13, 14]). Here, we focus on self as a conceptual representation, and do not directly address the experiential aspect. The view of the self we use here is similar to Thomas Metzinger’s unconscious self-models [15–17]. It is compatible with some formulations of predictive coding and free energy theories of self [6, 18–20], as well as with connectionist or memory-based models of self [21–24].

Information related to the self is processed and stored in a preferential manner. Self-related information is associated with better encoding than other-related information [9, 23, 25–29], and self-knowledge is organized in a more abstract way than knowledge about other people, which relies to greater extent on episodic recollection [30, 31]. Self-association has also a profound effect on perception, leading to facilitated processing of self-related stimuli, such as pictures of one’s own face [32–37]. A classic example of preferential self-processing in perception is the "cocktail party effect” [38]. During a noisy party, even when we are engaged in a conversation, we can easily hear our name in the otherwise unintelligible noise of other people's conversations.

Recent studies have shown that the effect of the self on perception can also be seen for arbitrary stimuli associated with the self. Sui, He and Humphreys [39] instructed their participants to learn arbitrary associations between geometrical shapes and three identities. For example, participants were instructed that “you [the participant] are a triangle, your best friend is a square, and a stranger is a circle”. In a perceptual matching task participants then judged whether pairs of stimuli presented together, a label and a shape, were matching or non-matching. Participants were faster and more accurate when judging correct pairings associated with self than correct pairings associated with friend or stranger. This result has been replicated in studies using other kinds of stimuli [40–43], including faces of unfamiliar people [44], avatars [42], and also stimuli processed by other sensory modalities [45]. Together, these results provide evidence that arbitrary stimuli that became associated with the self are processed preferentially. The aim of the present study was to investigate in more detail the time course of processing of these self-associated stimuli in order to determine whether activation of self-representation by stimuli associated with self will lead to a facilitation of ensuing stimuli even if these stimuli are not associated to self. This can be achieved with electrophysiological methods, such as EEG, and appropriately designed experimental paradigm.

Earlier EEG studies found that preferential processing of self-related stimuli is usually reflected in two event-related potentials (ERPs) in the EEG: the N2 and the P3 (for a review see: [46]), especially in studies concerned with face perception. The anterior N2 is a negative deflection occurring between 200 and 300ms after stimulus onset and it is considered an index of detection of novelty or mismatch, and of the need to exert cognitive control [47]. Several studies reported a decrease of the N2 in response to perceiving one’s own face compared to perceiving other faces [37, 48, 49]. The decrease in N2 for one’s own face can be interpreted as reflecting the fact that seeing one’s own face is less surprising than seeing other faces. This in turn may be a consequence of the fact that people are more familiar with their own face than with other people’s faces.

Perhaps the most robust finding in EEG research on preferential processing of the self is an increase in the P3 amplitude in response to self-related stimuli. The P3 is a positivity that occurs about 300 to 500ms after stimulus presentation. It is typically assumed that the P3 consists of two subcomponents: an earlier frontal one reflecting attentional processing of the perceived stimulus, and a later central-parietal one, which according to different theories reflects working memory processing, decision making, or response preparation [50–52]. The topology of the increased P3 amplitude for self-related stimuli varies across studies and doesn’t seem to follow a clear pattern. Perception of one’s own face led to an increased P3 at parietal sites in studies that used experimental tasks such as passive viewing [48] and recognition tasks ([34, 36, 53]). When participants were asked to respond to their own face but not another face or vice versa, the topography was more central [54]. When participants judged the orientation of their own and other faces the P3 increase was present at more frontal sites [37]. An enhanced P3 has also been observed for self-related stimuli other than one’s own face, such as one’s own name [34, 55–61], self-related possessive pronouns [62, 63], one’s own handwriting [64], and perceiving one’s own hand compared to a different hand [65].

In the present study, we investigated the time course of neural response to performing a matching between faces either associated or not associated with self to labels associated or not associated to self. Importantly, participants did not see their own faces. Rather, we established arbitrary associations to self with unfamiliar faces in the same way as [44]. Given prior research on self-prioritization, we expected that associating arbitrary faces with self would lead to a similar advantage in self-related processing as participants’ real faces, names, and other markers of self.

Faces were chosen, because they are strong markers of a person’s identity [66, 67] and because the neural mechanisms of face perception are well understood [68–70]. If prioritizing of self-relevant information depends on arbitrary associations between perceptual features and a self-concept [39] then the same modulation of ERP components observed in response to one’s face in previous research (smaller N2, bigger frontal or central-parietal P3) should be observed in response to an arbitrary face associated with self, corroborating behavioral results from [44]. If self-prioritizing occurs only for one’s actual face no differences should be expected between a self-associated face and faces associated with other identities.

We employed a similar matching task as Payne et al. [44] who asked participants to determine whether an arbitrary face corresponded to the label “you”, “friend”, or “stranger”. However, in the present study face and label were not presented simultaneously, as in [44], but sequentially, with a time interval of 1.5s between the presentation of face and verbal label. This enabled us to separate ERP components related to processing of the face, and ERP components related to processing of the label. To account for the possible role of the sequential order of presenting face and label, we conducted two experiments. In the first experiment the face was presented first and the task was to judge whether the label presented afterwards matched with the face. In the second experiment, the label was processed first and the task was to judge whether the face presented afterwards matched with the label. The participants had to judge if the second stimulus corresponded to the first one or not by pressing a specified key as quickly as possible.

Our first prediction was that self-prioritization effects should also occur when labels and arbitrary faces are presented sequentially rather than simultaneously like in [44]. Specifically, we predicted that RTs would be faster in matching trials where a face associated with self was followed by a label related to self (Experiment 1) or where a label related to self was followed by face associated with self (Experiment 2).

We expected that self-prioritization would also occur in non-matching trials. If the presentation of an initial self-related stimulus leads to sustained activation of a self-representation one should be faster to detect contradictory evidence than when the initial stimulus is not self-related. Furthermore, a self-representation should also be activated when a self-related stimulus (label in Experiment 1 and face in Experiment 2) follows a stimulus that is not related to self, speeding up RTs.

For the ERPs, we expected an increased amplitude of the P3 following presentation of stimuli associated with self, both for the first stimulus in the sequence (face in Experiment 1 and label in Experiment 2) and the second stimulus in the sequence (label in Experiment 1 and face in Experiment 2). An enhanced P3 in response to an arbitrary face associated with self would indicate enhanced attention and working memory activity in response to self-related stimuli. Moreover, we expected an effect of self-prioritization on the anterior N2 amplitude. It should be noted that, because the labels were not controlled for word length and familiarity, which are factors that are known to influence the N2 [71, 72], we only investigated the N2 that was elicited by face stimuli. We expected to find decreased amplitude of the anterior N2 following presentation of self-associated faces. A smaller N2 in response to an arbitrary face associated with self would indicate that the N2 effect observed for one’s real face does not reflect familiarity with one’s own face, but a different, more specific process related to preferential processing of faces associated with self. The lack of an N2 effect for self-associated face would indicate that this effect is driven by familiarity.

## Experiment 1

### Methods

#### Ethics statement

The study has been approved by the Ethical Research Committee of Central European University. All participants gave informed consent in written form. All figures were prepared by the first author of the manuscript and were not published before. Figures contain photographs of three individuals. The individuals from the photographs have given written informed consent (as outlined in PLOS consent form) to publish these photographs. This ethics statement applies to both experiment 1 and 2.

#### Participants

Nineteen people participated in the study, but one person was excluded due to the low quality of the EEG data obtained. The age range of the remaining eighteen participants was between 20 and 30 years (*M* = 25.4, *SD* = 3.03). Half of them were female and all of them were right-handed. All participants had normal or corrected-to-normal vision.

#### Apparatus, stimuli, and procedure

The experiment consisted of two parts: in one part, the face stimuli were of the same gender as the participant, and in the second part of the opposite gender. Except the gender of the face stimuli, the experimental procedure was identical, therefore the description of the task applies to both parts. The experimental task was divided into three phases (learning, test, and matching task) and each of them was performed on a PC computer within one experimental script using E-Prime 2.0 software (Psychology Software Tools, Inc., Sharpburg, PA, USA). The purpose of the learning phase was to teach participants about the identity of three faces. They were presented sequentially with one of three possible descriptions: "Remember that this face is:", and then either "You", "Friend", or "Stranger". Each face with an accompanying label was presented for 20 seconds and the order of presentation was random.

The testing phase served to ensure that participants had acquired the associations between verbal labels and faces. It consisted of 24 trials during which participants were presented with a picture of one of the faces centered on the screen, together with two labels, one on the left and one on the right. The labels could be "You", "Friend", or "Stranger". Participants indicated which label matched the face using two keys on the keyboard. Both the face and the labels stayed on the screen until participants responded or until 5 seconds passed. Each face and each label was presented an equal number of times and all possible combinations were counterbalanced. After pressing a key, participants were receiving feedback, which was either "Correct" in blue, "Incorrect" in red or "No response" in red in case of no key press. The feedback was visible for 250ms. If the participants responded correctly in at least 90% of the trials, they could proceed to the next phase. If not, they repeated the learning phase and the test until they had acquired the association between faces and labels.

**Fig 1:**
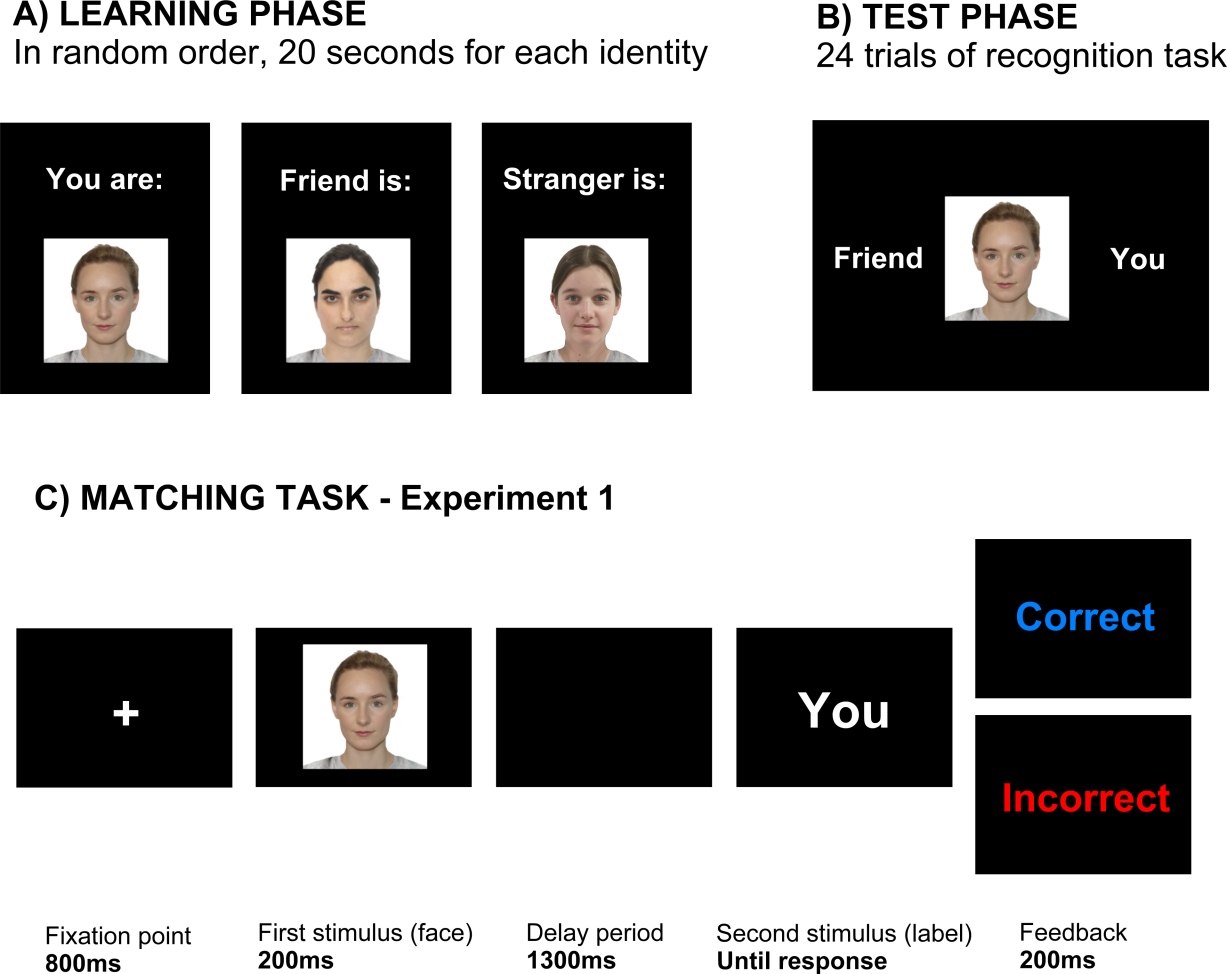
The procedure of experiment 1. (A) Participants started with the learning phase during which they were presented with three faces together with their identities. The faces were presented sequentially in random order for 20 seconds each. (B) Following the learning phase, participants completed a test assessing whether they had learned to correctly assign labels to faces. In this test they were asked to choose which of two labels corresponded to a particular face. (C) The main task was a matching task during which EEG was recorded. Each trial started with a fixation cross followed by a short presentation of a face. After 1300ms of a delay period a label was displayed and stayed on the screen until the participant responded. The photographs are for illustration purposes, they were not the faces used in the task. The individuals from the photographs have given written informed consent (as outlined in PLOS consent form) to publish these photographs.

In the main phase of the experiment participants performed a matching task and their RTs and their EEG was recorded. Participants performed four blocks of 84 trials amounting to 336 trials (thus leading to 672 trials in total for both genders of the faces). Each trial started with a fixation cross visible for 800ms, followed by a 200ms presentation of one of three faces (Fig 1) and a blank screen for 1300ms. It was then followed by the presentation of one of the labels. The task was to judge whether the label corresponded to the earlier presented face. The judgment was executed by pressing one of two keys using a standard keyboard. Each key was pressed with a different hand. The mapping of the keys was kept constant throughout the experiment but key mapping was counterbalanced across participants. The label stayed on the screen until the response was made and then was replaced with feedback information for 200ms. The time to respond was limited to 3000ms. The duration of the inter-trial interval randomly varied between 800ms and 1800ms.

The trials in each block followed a random order such that each of the three faces was presented an equal number of times (28 within a block) and so that the label corresponded to the face 50% of the time. When the label did not correspond to the face, there was an equal probability to see each of the two incongruent labels. This ensured that participants were not biased to respond "Incorrect", as would have been the case if there was an equal probability of seeing each of the three labels after any given face.

The experiment was performed on a PC with a 24'' widescreen LED monitor. The labels and other written stimuli were white, presented on a black background. The size of the fixation cross was 0.6°x0.6° and the width of the labels was between 2° and 4.4°. Pictures of the faces were taken from the Chicago Face Database [73]. Three female and three male faces were chosen from among the available Caucasian faces with neutral facial expression (female faces: WF-209, WF-211, WF-233, male faces: WM-003, WM-004, WM-029). The full size of the pictures in the experiment was 8°x8°.The size of the face alone was 3.8°x5.3°. The Chicago Face Database comes together with ratings of each face on several scales (e.g. attractiveness, trustworthiness, surprise, masculinity, sadness, etc.). The faces were chosen in such a way to make them as similar as possible on the attractiveness scale, but also not too different on the other scales. The matching of faces to identities was counterbalanced across participants.

#### Data acquisition

Behavioral data was recorded from participants' key presses using a standard computer keyboard. EEG was recorded continuously throughout the experiment using a 10–20 cap (EasyCap, Brain Products GmbH, Germany) with 63 electrodes relative to an offline average mastoid reference. Eye movements were monitored using two pairs of EOG electrodes. One pair was positioned lateral to the left and right eye and enabled detection of horizontal eye movements. The second pair was positioned above and below the left eye, and enabled detection of vertical eye movements. Electrode impedance was kept below 10kΩ. EEG and EOG signals were amplified using a band-pass of 0-250Hz by two BrainAmp DC amplifiers (Brain Products GmbH, Germany) and sampled at 500Hz.

#### Data processing and analysis

Incorrect trials were not included in the analysis. Moreover, trials with correct responses which were faster than 200ms were excluded based on the assumption that they were too fast to reflect perceptual matching. Trials with correct responses which were slower than 1600ms were also excluded to reduce the influence of random outliers caused by delay in response. Based on these three criteria 2.2% (*SD* = 2.1%) of the trials were removed. Processing and analysis of EEG data was performed offline using Brain Vision Analyzer 2.1.0 (Brain Products GmbH, Germany). Data was filtered using a low cut-off filter of 0.01Hz (24 dB/octave) and a high cut-off filter of 50 Hz (24 dB/octave) to remove the influence of slow drifts and excessive high-frequency noise. Additionally, a notch filter at 50Hz was used to remove possible artifacts caused by electrical devices present in the room. The participants were instructed to blink only during the inter-trial intervals, but in order to reduce the influence of eye movements which occurred nevertheless during the analyzed period, an Ocular Correction procedure was applied to the segmented data using the Gratton & Coles algorithm [74], as implemented in Brain Vision Analyzer 2. After Ocular Correction, the data were visually inspected in order to ensure that the algorithm did not lead to spurious results.

For analysis of the stimulus-locked potentials in response to the face, data was segmented offline into epochs starting 300ms before the stimulus onset and ending 3300ms after stimulus onset, and baseline-corrected relative to the last 200ms before stimulus (i.e. face) onset. For the stimulus-locked potentials caused by the label, data was segmented into epochs starting 1750ms before the onset of the cue and ending 1750ms after the onset of the cue, and baseline-corrected relative to the last 100ms before stimulus (i.e. label) onset. We selected a shorted baseline here in order to reduce the influence of any possible differences in readiness potential steepness between the conditions. Artifact rejection was performed automatically by removing the whole segments which either showed more than 120μV changes within the segment (for five participants in experiment 1this threshold was increased to 150μV because of excessive noise in the signal) or exhibited activity lower than 0.5μV for at least 100ms leading to the removal of on average 6.8% of the trials (*SD* = 6.4%).

The results were analyzed with a one-way repeated measures ANOVA. If Mauchly's test indicated violation of assumption of sphericity, Greenhouse-Geisser's correction was applied. If the main effect of the ANOVA was significant, two planned Helmert contrasts were used to assess the significance of differences between individual levels (see [75] for explanation). The first contrast compared self against an average of friend and stranger, and served as the crucial test for the self-prioritization effect. The second contrast compared friend against stranger, and provided additional information about the pattern of the data, i.e. whether association with a friend leads to a friend-bias when compared to stranger. The same procedure was used in previous studies on self-prioritization by [42] and [44].

**Fig 2:**
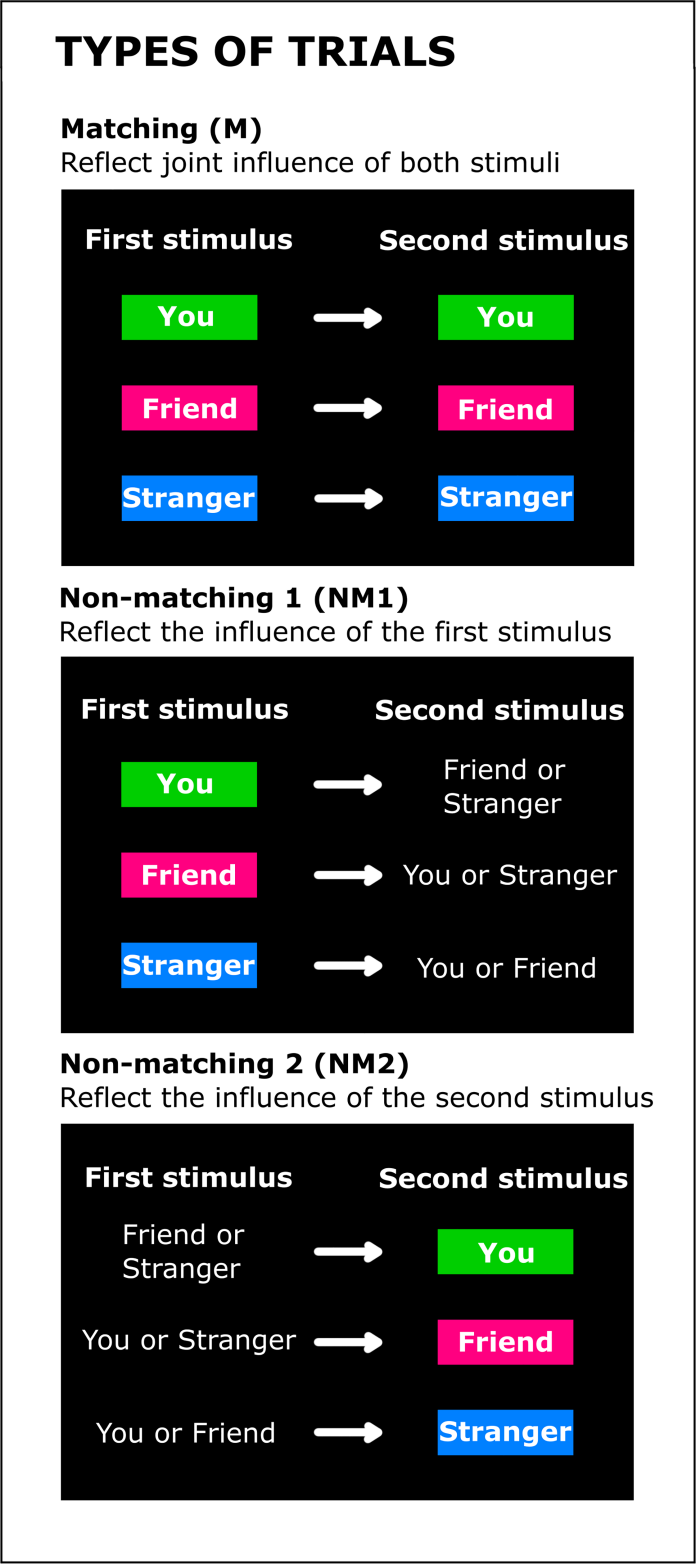
Three types of trials used to analyze the data from both experiments. In matching trials face and label corresponded with one another. In non-matching trials they did not correspond. There are two ways to categorize non-matches. One way (non-matching 1) is to take the first stimulus as a reference and to determine how the second stimulus is mismatching. This allows one to assess the influence of expectations induced by different stimuli (you, friend, stranger) on RTs. The other way (non-matching 2) is to take the second stimulus as a reference and to determine whether the first stimulus was mismatching. This allows one to assess the influence of different stimuli presented second (you, friend, stranger) on the participant’s RTs.

The data were analyzed separately for the matching and non-matching trials (Fig 2). Non-matching trials can be analyzed in two ways. One way is to take the first stimulus as the reference for a mismatch: For instance, if the first stimulus is a friend-associated face then the label "You" (referring to self) or "Stranger" can create a mismatch with regard to the first stimulus. We will refer to this categorization as "Non-Matching 1" (NM1). Categorizing trials in this way reflects the influence of the first stimulus on RTs. Another way is to take the second stimulus as the reference. For example, in trials were the second stimulus was the label "You", mismatches could occur because the label was preceded by a friend-associated or a stranger-associated face. We will refer to this categorization as "Non-Matching 2" (NM2). NM1 and NM2 averages are calculated using the same data (all mismatching trials), therefore their grand averages are the same.

The use of EEG allowed us to separately investigate cognitive processes related to face processing (first stimulus) and related to label processing (second stimulus). The first ERP of interest elicited by presentation of faces was the N2, which was quantified by pooling the mean activity between 250 and 350ms after face onset from electrodes AF3, AFz and AF4. The electrodes were chosen to reflect the anterior N2 described in [47]). The N2 was followed by a prolonged positivity, peaking around 700ms after face onset. Closer inspection of the data revealed that there was an effect of self-prioritization over the same frontal area as in the case of the N2. This positivity, which from now on will be referred to as late frontal positivity (LFP) was quantified by pooling the mean activity between 450 and 700ms after face onset from electrodes AF3, AFz and AF4. With respect to label processing, we focused our analysis on the central-parietal P3, which we quantified by pooling the mean activity between 250 and 400ms after label onset from electrodes Cz, CPz and Pz (reflecting standard location of the central-parietal P3 [50]). The time intervals of the analysis were selected based on the grand average topographies.

### Results

#### Behavioral data

Analysis of the behavioral data was conducted separately for trials in which the label matched the preceding face and for trials in which the label did not match the preceding face. Because accuracy was high (*M* = 97.8%, *SD* = 2.1%) we focused only on the analysis of RTs for correct responses.

**Fig 3:**
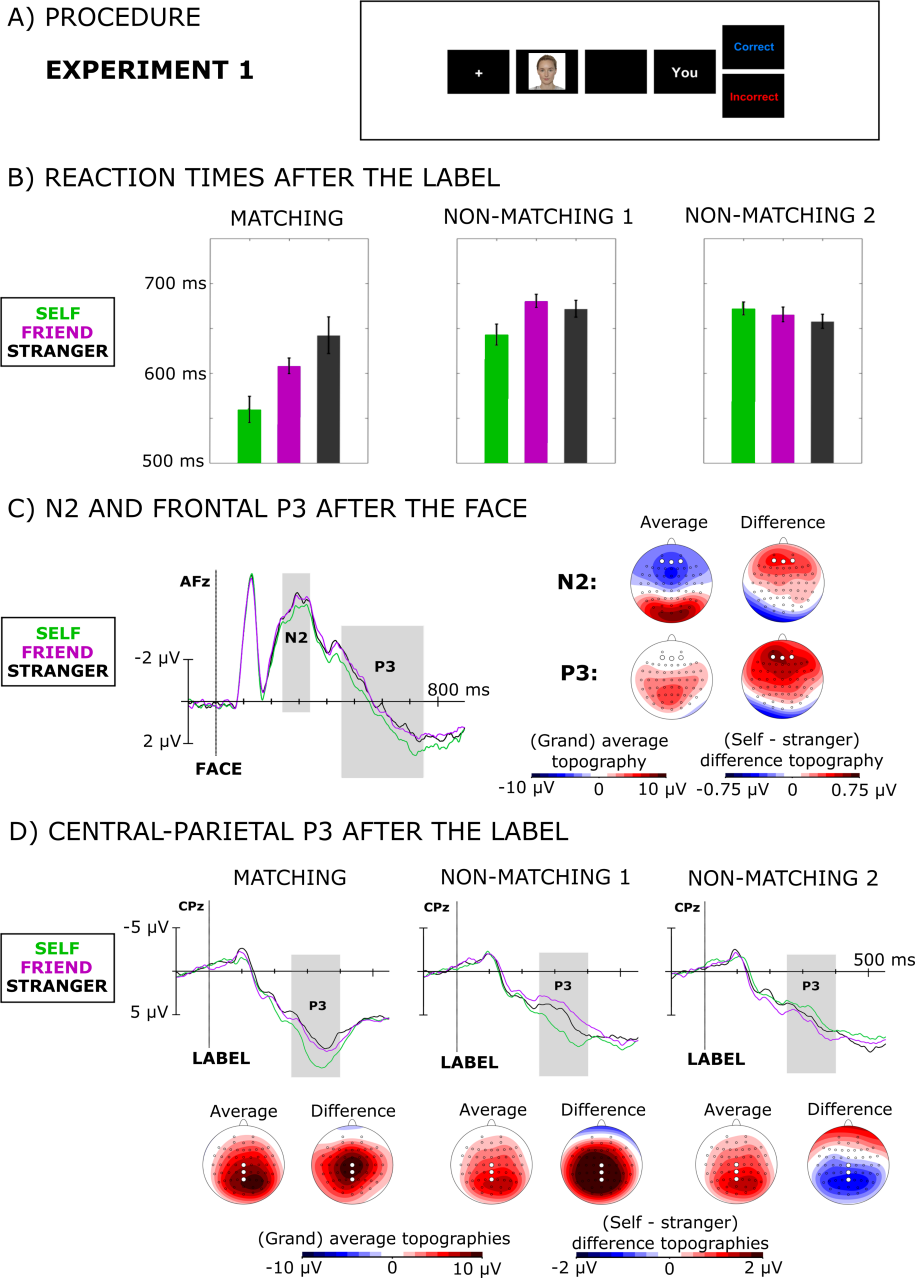
The results of experiment 1. (A) A single trial from experiment 1 illustrating the procedure. (B) Reaction times separately for (from left to right) matching, non-matching 1 (NM1), and non-matching 2 (NM2) trials (see Fig 2 for explanation of how NM1 and NM2 were derived) for self (green), friend (purple), and stranger (black). (C) Event-related potentials following presentation of the face, including the time course of the N2 (between 240 and 340ms) and the late frontal positivity (450-750ms) at electrode AFz (left panel), and corresponding average of all conditions voltage topographies and voltage topographies of the difference between the self-condition and the friend/stranger conditions (right panel). (D) Average of all conditions topographies and grand-averaged stimulus-locked waveforms of the central-parietal P3 for self, friend, and stranger at electrode CPz, separately for matching trials and two types of mismatching trials (NM1 and NM2). The individual from the photograph has given written informed consent (as outlined in PLOS consent form) to publish this photograph.

The left panel in Fig 3B shows RTs for matching trials. Matching trials showed a clear self-prioritization effect (main effect: *F*(2,34) = 18.75, *p*<0.001 Greenhouse-Geisser corrected, partial *η*^*2*^ = 0.52). Reaction times were shorter if an arbitrary face was associated with self than when it was associated with friend or stranger (*F*(1,17) = 34.3, *p*<0.001). The difference between friend and stranger was also significant (*F*(1,17) = 5.8, *p* = 0.028) with shorter RTs for friend than stranger.

The middle and right panels in Fig 3B show RTs for non-matching trials (NM1 and NM2). A one-way repeated measures ANOVA showed a main effect for NM1 (*F*(2,34) = 10.62, *p*<0.001, partial *η*^*2*^ = 0.38), but not for NM2 (*F*(2,34) = 2.17, *p* = 0.13). Helmert contrasts for NM1 led to similar results as in the matching trials. Reactions were significantly faster for self than for friend and stranger (*F*(1,17) = 13.7, *p* = 0.002). There was no significant difference between friend and stranger (*F*(1,17) = 2.0, *p* = 0.17).

#### N2 and late frontal positivity evoked by the first stimulus (face)

Presentation of a self-associated face led to modulation of the anterior N2 between 250 and 350ms (*F*(2,34) = 3.40, *p* = 0.045, partial *η*^*2*^ = 0.17). In line with previous research, the amplitude of the N2 was smaller for self than for friend or stranger (*F*(1,17) = 5.5, *p* = 0.031). There was no difference between friend and stranger (*F*(1,17) = 0.1, *p* = 0.73). In addition, it led to a prolonged increase in amplitude of the late frontal positivity between 450 and 750ms (*F*(2,34) = 6.12, *p* = 0.005, partial *η*^*2*^ = 0.27), compared to faces associated with other identities (*F*(1,17) = 10.1, *p* = 0.005). The difference between friend and stranger was not significant (*F*(1,17) = 0.1, *p* = 0.76).

#### Central-parietal P3 evoked by the second stimulus (label)

As expected, a central-parietal P3 was also present after presentation of verbal labels. Interestingly, self-related differences depended on the relation between the first and the second stimulus (Fig 2). For the **matching** trials, representing the joint influence of face and label, there was a significant main effect of identity over the central-parietal cortex between 250 and 400ms (*F*(2,34) = 9.4, *p* = 0.001, partial *η*^*2*^ = 0.36). Self-related matching pairs elicited stronger positivity than pairs identified with friend and stranger (*F*(1,17) = 12.7, *p* = 0.002). There was no difference between friend and stranger (*F*(1,17) = 1.95, *p* = 0.18).

The P3 amplitude for non-matching trials followed a similar pattern as the results for reaction times. There was a significant effect in the Non-Matching 1 condition (*F*(2,34) = 16.92, *p*<0.001, partial *η*^*2*^ = 0.50). When the first stimulus was a face associated with self, the central-parietal P3 was stronger than when the initially presented face was associated with another identity (*F*(1,17) = 19.95, *p*<0.001). Moreover, there was a difference between friend and stranger, with stranger as a first cue leading to stronger amplitude of the P3 than friend (*F*(1,17) = 8.4, *p* = 0.010).

Interestingly, the Non-Matching 2 condition led to the reversed pattern compared to the matching and NM1.There was a significant main effect of identity (*F*(2,34) = 4.53, *p* = 0.018, partial *η*^*2*^ = 0.21), but the differences went in the opposite direction than in the matching and NM1 conditions. The P3 was smaller for a mismatching self-label than for the other labels (*F*(1,17) = 4.8, *p* = 0.042). The difference between friend and stranger did not reach significance (*F*(1,17) = 3.9, *p* = 0.064).

### Discussion

Experiment 1 investigated the time course of self-prioritization effects using a task that enabled us to disentangle the processing of faces and labels associated with them. In line with previous research using perceptual matching of synchronously presented stimuli, we found a robust self-prioritization effect on reaction times in matching trials (when the identity of a label corresponded to the identity of a face). We found the same effect for non-matching trials, but only when the initially presented face was associated with self (NM1 trials). There was no effect of subsequently presented self-related verbal labels (NM2 trials).

The EEG results provided further insights into the processes underlying self-prioritization. They showed that perceiving an unfamiliar face associated with self, modulated the amplitude of two ERPs that have previously been shown to be modulated by perception of one’s own face [46]. First, perceiving an arbitrary face associated with self, decreased the amplitude of the anterior N2 between 250 and 350ms. [37, 48, 49] report similar effects for perceiving one’s own real face. Interestingly, this N2 effect was present even though the faces associated with each identity were equally unfamiliar before the experiment and the assignment of faces to self was counterbalanced across participants. The amplitude of the N2 is argued to reflect novelty [47] and, therefore, the decreased amplitude for self-associated faces indicates that associating an arbitrary face to self can lead to a quick increase in familiarity for this face. Importantly, self-associated faces were presented equally often as faces associated with friend and stranger. Thus, the differences in N2 amplitude cannot be due to higher frequency of presenting the self-associated face. It seems that any self-associated face, including arbitrary faces associated to self, is prioritized in perception. This finding is in line with Bayesian models of perception and cognition (e.g. [76, 77]) where self-prioritization could be described as increasing the strength of perceptual priors in a similar way as the visual cortex is tuned for more probable upcoming scenes [78, 79].

The N2 was followed by a long-lasting positivity that had larger amplitude over frontal areas when faces were associated to self. We refer to this ERP as late frontal positivity (LFP). Similar positivities have been described in prior research as long-latency positive component [37] or as late slow wave [58, 60, 80]. The fact that the LFP peaks quite late (i.e. ~700 after stimulus onset, see Fig 3C) may be caused by the difficulty of processing previously unfamiliar faces. The amplitude increase of the LFP in the present study resembles the well-established effect of the self on the P3, which is often present in frontal sites (e.g. [37, 49, 64, 65]).

As predicted, we also observed an increased amplitude of the P3 following self–associated verbal labels. For labels, the difference between the conditions was present at the central-parietal electrodes. Similar results were obtained in studies that investigated perception of one’s own name [55, 57]. Interestingly, the pattern of results followed the one observed for reaction times, i.e. the central-parietal P3 was stronger for self than for friend/stranger in the matching and NM1 trials. This effect was absent in NM2. This indicates that both reaction times and the central-parietal P3 reflect activation of a self-representation that facilitates processing of ensuing information that is potentially relevant for self.

In two instances we found a significant difference between friend and stranger, i.e. faster RTs for friend than stranger in the matching trials, and increased amplitude of the central-parietal P3 for stranger than friend in the NM1 trials. Although the former effect is consistent with expected prioritization of friend than stranger-associated stimuli (e.g. [39]), the latter goes in the opposite direction. Taken together with the fact that we did not find other differences, the data does not allow us to draw firm conclusions about differences in processing between friend and stranger-related information.

## Experiment 2

The self-prioritization effects observed in Experiment 1 can be explained in two ways. First, there may be a specific link between faces and a self-representation that causes faster processing of ensuing self-relevant information. If this is the case, then faces presented after a self-related verbal label should lead to self-prioritization effects in matching trials and NM2 but not in NM1. Alternatively, any self-related stimulus may activate a self-representation, which would then enhance processing of subsequent stimuli. In this case, the results from experiment 1 should replicate when verbal labels are followed by faces: We should find self-prioritization effects in matching and NM1 trials, but not in NM2 trials. The second experiment tested these hypotheses. Participants were first presented with a verbal label, which could refer to self ("You") or not ("Friend", "Stranger"), and were then asked to judge if the subsequent face matched the label.

### Methods

#### Participants

Twenty participants were tested. Two were excluded from the analysis because of poor quality of the EEG data (more than 40% of trials had to be excluded due to EEG noise). Out of the remaining eighteen participants 9 were females. The range of age of the participants was between 18 and 39 years (*M* = 25.9, *SD* = 5.47). Two participants were left-handed, but their results did not differ from the rest of the group. All participants had normal or corrected-to-normal vision. The study has been approved by the Ethical Research Committee of Central European University. All participants gave informed consent in written form.

#### Procedure

**Fig 4:**
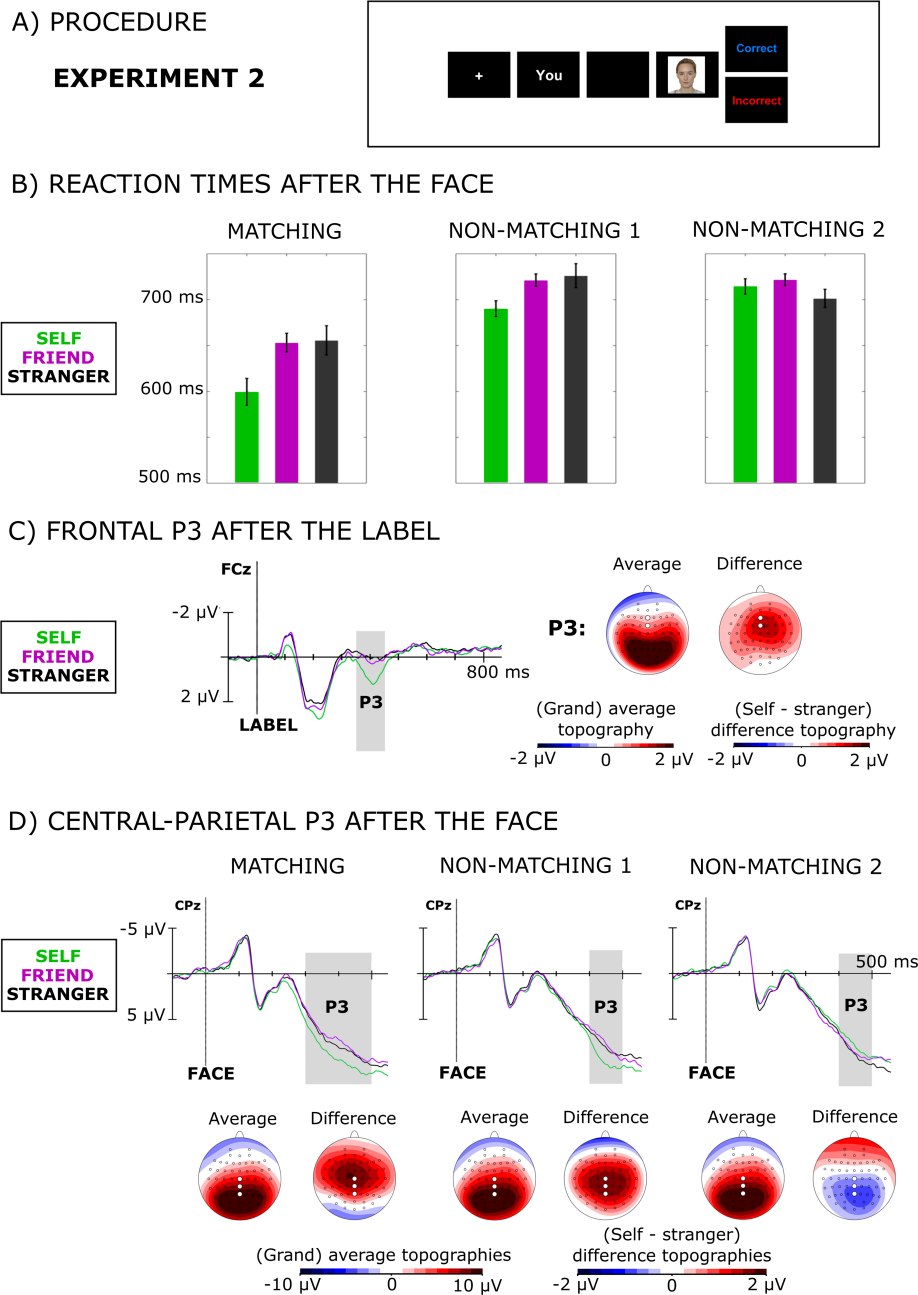
The results of experiment 2. (A) A single trial from experiment 2 illustrating the procedure. (B) Reaction times separately for (from left to right) matching, non-matching 1 (NM1), and non-matching 2 (NM2) trials (see Fig 2 for explanation of how NM1 and NM2 were derived) for self (green), friend (purple), and stranger (black). (C) The time course of the frontal P3 (350-450ms) following presentation of a label at electrode FCz (left panel), and corresponding average of all conditions voltage topographies and voltage topographies of the difference between the self-condition and the friend/stranger conditions (right panel). (D) Average of all conditions topographies and grand-averaged stimulus-locked waveforms of the central-parietal P3 after presentation of the face for self, friend, and stranger at electrode CPz, separately for matching trials and two types of mismatching trials (NM1 and NM2). The individual from the photograph has given written informed consent (as outlined in PLOS consent form) to publish this photograph.

The procedure was the same as in Experiment 1, with one exception: the order of the stimuli was reversed (Fig 4A). The first stimulus was a label ("You", "Friend", "Stranger"), and the second stimulus was a picture of a face.

#### Data processing and analysis

The behavioral analyses were conducted in the same way as those in Experiment 1. On average 2.3% (*SD* = 1.6%) of the trials were removed due to participants’ mistakes or reaction times shorter than 200ms or longer than 1600ms. With regards to EEG analysis and similar to Experiment 1, the presentation of labels elicited a central-parietal positivity, but the effect of self-prioritization was observed over frontal sites. The time course of this (transient) frontal positivity resembles the frontal P3 and was quantified by pooling the mean activity between 350 and 450ms from electrodes Fz and FCz. The selection of these two electrodes was based on visual inspection of the grand average waveforms across all conditions, which demonstrated a clear positive peak in the P3 time range (between 350 and 450ms) and is also consistent with the existing literature. The presentation of faces elicited a central-parietal P3, which was quantified by pooling the mean activity from the same electrodes as in Experiment 1 (i.e. Cz, CPz and CPz) to make our results as comparable as possible. The intervals of analyses were 300 to 500ms for the matching trials and 400 to 500ms for the non-matching trials. The differences in the selection of the interval of interest reflect latency differences in the P3, which are most likely related to the different levels of difficulty between the matching and non-matching conditions [50, 81].

An analysis of the N2 following the faces is presented in the supplementary materials. Similar to Experiment 1, four participants had artifact rejection done with threshold increased to 150μV due to increased noise in the signal. The artifact rejection procedure led to rejection of on average 7.8% of the trials (*SD* = 6.1%).

### Results

#### Behavioral data

Accuracy was very high and was therefore not further analyzed (*M* = 97.7%, *SD* = 1.6%). The left panel of Fig 4B shows the RTs for matching trials. There was a significant main effect (*F*(2,34) = 10.10, *p*<0.001, partial *η*^*2*^ = 0.37) because reactions were significantly faster for self than for friend and stranger related pairs (*F*(1,17) = 16.4, *p* = 0.001). There was no difference between friend and stranger (*F*(1,17) = 0.05, *p* = 0.83).

The middle and right panel of Fig 4B show the results for mismatching trials categorized according to reflect the influence of first (NM1) or second stimulus (NM2), see Fig 2. NM1 reflects the influence of the initially presented label and showed a significant main effect (*F*(2,34) = 8.41, *p* = 0.006 Greenhouse-Geisser corrected, partial *η*^*2*^ = 0.33). Reaction times were shorter when the first stimulus was related to self than when it was related to friend or stranger (*F*(1,17) = 21.9, *p*<0.001). There was no difference between friend and stranger (*F*(1,17) = 0.13, *p* = 0.72). NM2 reflects the influence of the face presented after the label. There was a significant main effect (*F*(2,34) = 4.16, *p* = 0.024, partial *η*^*2*^ = 0.20). However, the main effect was not due to differences in RT between self-associated faces and friend/stranger faces (*F*(1,17) = 0.28, *p* = 0.6). Rather, it was due to a significant difference between friend and stranger (*F*(1,17) = 8.41, *p* = 0.01) with faster reaction times for stranger.

#### Frontal P3 evoked by the first stimulus (label)

A repeated measures ANOVA on pooled activity from electrodes Fz and FCz between 350 and 450ms showed a main effect on P3 amplitude (*F*(2,34) = 19.85, *p*<0.001, partial *η*^*2*^ = 0.54), because the P3 was larger following presentation of self-related labels (*F*(1,17) = 36.4, *p*<0.001) than the labels referring to friend or stranger. There was no difference in the P3 amplitude following the labels “Friend” and “Stranger” (*F*(1,17) = 2.1, *p* = 0.168).

#### Central-parietal P3 evoked by the second stimulus (face)

In matching trials there was a strong significant main effect of identity on P3 amplitude between 300 and 500ms post stimulus (*F*(2,34) = 12.08, *p*<0.001, partial *η*^*2*^ = 0.42). There was a stronger positive deflection for faces associated with self than for faces associated with friend or stranger *F*(1,17) = 18.7, (*p*<0.001). The difference between the two latter faces was not significant (*F*(1,17) = 1.4, *p* = 0.25).

There was also a significant effect in the non-matching 1 trials (*F*(2,34) = 6.56, *p* = 0.010 Greenhouse-Geisser corrected, partial *η*^*2*^ = 0.28) with the same pattern of differences, but beginning later, around 400ms after the stimulus presentation. Faces that had been preceded by the self-related label "You" led to stronger central-parietal P3 than faces that were preceded by labels related to "Friend" and "Stranger" (*F*(1,17) = 13.8, *p* = 0.002). There was no difference between the latter two conditions (*F*(1,17) = 0.2, *p* = 0.66). In the NM2 condition, the posterior P3 did not differ between different conditions (main effect: *F*(2,34) = 1.40, *p* = 0.26) in the time window between 400 and 500ms.

### Discussion

Experiment 2 supported and extended the results from experiment 1. It replicated the pattern of results for reaction times, pointing to the importance of self-reference of the first stimulus in eliciting self-prioritization. The self-prioritization effect was present when the first stimulus was associated with self (in this case the label “You”), whereas the identity of the second stimulus did not play a significant role. This result indicates that there is no special link between face processing and activating conceptual self-representation. Self-related labels, and presumably other type of self-related information, seem to have the same access to the conceptual self as faces. What does matter is only the position in the task–the response is faster if the first, but not the second, stimulus is self-related.

The EEG results were also consistent with the EEG results of Experiment 1. The initially presented label evoked a frontal P3, which was larger when the label was self-related and did not differ between “Friend” and “Stranger”. This result is in accord with previous studies on processing of one’s own name and self-referential personal pronouns [59–61, 82]. The face following the label evoked a central-parietal P3 that was larger when the initial label was self-related. Surprisingly, self-relatedness of the face, in response to which the P3 occurred, had no effect on the amplitude of this component. Taken together, the behavioral and EEG analyses indicate that self-prioritization occurs because initially encountered information activates a conceptual self-representation and facilitates processing of ensuing stimuli that are potentially self-related.

It should be mentioned that the analysis of the N2 following faces replicated the findings from the first experiment (smaller amplitude of N2 for self-associated face than other faces). Because this result is not crucial for our main conclusions and because this analysis required an additional transformation of the data due to the design of Experiment 2, we report these results in the supplementary materials (S1 Text).

## General discussion

There are two main findings in the results of the present experiments. First, we showed that associating previously unknown faces to self leads to similar differences in self-related processing as faces or labels that have a long history of having a strong association to self, e.g. when one sees one’s own face or is addressed as “You”. These differences were not just evident in faster reactions for faces and labels associated to self, but also in the modulation of the amplitude of several ERPs that are considered to reflect processing of self-related information.

The second finding was that the self-relevance of initially encountered information has a decisive role in the processing of subsequent information. Specifically, self-associated stimuli facilitated processing of subsequent stimuli, irrespectively of whether these stimuli were associated with self. In other words, when the first stimulus was not associated with self, there was no facilitation in the processing of the second stimulus even if it had an intrinsic association with self. These results extend the findings of previous studies demonstrating that stimuli, such as geometrical shapes or unknown faces, can be arbitrarily associated with the self after only a brief period of exposure to them [39, 44]. It suggests that any type of self-related stimuli can activate a conceptual self-representation, which in turn facilitates subsequent information processing. The fact that the pattern of results across our two studies was the same regardless of whether the first stimulus was a familiar linguistic label or an unfamiliar face, suggests that the self-representation causing prioritization is an abstract self-concept, self-representation at the semantic level. It is highly unlikely that prioritization was caused by lower-level systems based on a sensory body representation.

The sequential design of our matching task enabled us to separate the processes that occurred for two consecutive stimuli in the matching task and to thereby better understand the time course of self-prioritization. Both experiments showed that self-relevance of the first stimulus modulated the magnitude of ERPs at the frontal sites. These effects are in line with previous studies on self-referential processing. Perception of the self-related label “you” as the first stimulus (in experiment 2) led to increased amplitude of the P3 around 400ms after the onset of the stimulus. This replicates previous results on processing of personal pronouns [62, 63] and own name [34, 53, 59, 60], and provides further evidence that self-related words are processed preferentially. The preference may be caused by involuntary attentional orienting and stimulus categorization processes [50, 83].

Importantly, perception of self-associated faces led to the same modulation of ERPs as perception of one’s own face, i.e. reduced anterior N2 around 200-300ms (similar to: [37, 48, 49]) and increased late frontal positivity following the N2 (as in: [34, 37, 49]. The N2 effect in our study cannot be attributed to familiarity, because all faces were equally unfamiliar. Instead, it may be attributed to predictive processes preparing the visual system to perceive any kind of self-associated information including faces. If the system is more prepared to perceive self-associated faces then appearance of the face is less surprising, which is reflected in a smaller amplitude of the anterior N2 [47].

The N2 was followed by a prolonged increase of amplitude of the late frontal positivity. There are two ways in which the relation between the N2 and the LFP can be interpreted. First, both may reflect one and the same process manifested by an increased anterior positivity starting around 250ms post stimulus. In this case, the LFP may be considered as a late P3a, as described by [50], and may reflect increased attentional orienting towards a previously unknown face that has been categorized as related to self. The N2 would reflect the onset of the P3a and the LFP would just become its consequence. Alternatively, it is likely that the LFP may constitute a separate component from the N2. The second interpretation is supported by studies showing that the N2 is independent of subsequent frontal positivities [47]. According to this interpretation, smaller amplitude of the N2 may reflect reduced surprise, while the LFP may be a correlate of later processing stages, possibly related to preparing for the appearance of the second stimulus.

Surprisingly, in both experiments, the amplitude of the central-parietal P3 did not depend on the association to the self of the stimulus that elicited the P3, but instead on self-association of the preceding stimulus, regardless of whether this preceding stimulus was a label or a previously unknown face. Regarding the functional significance of the central-parietal P3, a widely-held view is that it reflects working memory processing (for a review: [50]). Accordingly, the enhancement of the central-parietal P3 after a self-relevant first stimulus may reflect increased activation of self-relevant information in working memory, leading to a speed up of matching judgments. However, more recent views on the central-parietal P3 propose that it may reflect a decision making processes in response to motivationally significant events [51] or activation of well-established stimulus-response links [52]. In this line of reasoning, the enhanced central-parietal P3 may not reflect activation in working memory, but rather a motivational bias that enhances decision making that involves self-related information, or a more robust link between a self-relevant stimulus and the required response.

Taken together, our results indicate that it is easy to associate information to self even if this contradicts well established associations, such as when an unfamiliar face is associated to self, and that self-relevance of information matters most when it is encountered early in a processing sequence. These results may have implications for other social phenomena, such as the ease with which people associate themselves with arbitrary in-groups thereby excluding arbitrary out-groups [84–86]. Group membership can be understood as relating a representation of a group to a self-representation. Thus we expect that group membership may produce similar prioritization effects as the ones found in the present study because stimuli related to the in-group will be processed preferentially to the ones related to the out-group. In fact, there is some recent evidence that this is the case [43]. Especially strong effects can be expected when group membership will be based on political beliefs, as suggested by the fact that they can form an especially rigid and important part of personal identity [87, 88].

Our study also provokes several questions within the domain of science of self. First, one may wonder to what extent the effect found in our study relates to lower-level embodied self-representations. A recent study by Payne et al. [44] using a similar self-prioritization task with unfamiliar faces found self-prioritization effect for self-associated face in a perceptual matching task (which reflects influence of self-association on the conceptual self-representation), while it did not find effects of arbitrary self-associations on multisensory representation of one’s real face, as measured by enfacement illusion. It suggests that these types of representations may be independent, although future research is needed to resolve this issue.

Second, our results can be discussed in relation to the issue of whether self-related effects emerge in tasks requiring either implicit or explicit access to self-representation. This can be assessed using tasks in which self-association is task relevant, or task irrelevant. In regard to this issue our results provide evidence of strong self-related effects in an explicit context (cf. [89]). There is separate evidence of effects (facilitated visual recognition) that exclusively appear in implicit task and not in explicit task. These studies typically address first-person perspective perceptual representations of one’s body [89–92], although similar effects have been also found in task involving implicit self-recognition of face-parts (eyes, mouth, nose) [90, 93], which were (by necessity) perceived from the third-person perspective. This raises the question to what extent tasks using implicit and explicit self-recognition may lead to different results when used to investigate different kinds of self-representations (e.g. conceptual/semantic versus sensory/body representations), and to a more general question: to what extent self-representations are distinct cognitive modules.

The third issue is the relationship between self-prioritization effect and conscious awareness. According to several philosophical [13, 94, 95] and scientific theories [96–98], conscious awareness is underpinned by the representational structure of the mind. As such, conceptual representations of the self, such as the one investigated in our study, may underpin the conscious experience of “me-ness” or “mine-ness”. Although our study did not investigate the subjective level of identification with a self-associated face, nor awareness of self-prioritization effect, these two issues pose interesting venues for future studies. Several recent studies approached the issue of modulation of self-related effects by conscious access and led to a mixed pattern of results. Macrea et al. [99] reported that self-associated information enters consciousness earlier than other information in continuous flash suppression task, while Stein et al. [100] reported no effect. Moreover, Tacikowski et al. [10] in an fMRI study show that partially different brain systems are responsible for processing self-related information on the conscious and unconscious level. All these studies suggest that there may be a complicated interaction between consciousness and the conceptual self, which needs to be further investigated.

To conclude, in line with Sui & Humphreys claims ([39], also: [4, 101]), our study suggests that arbitrary information related to self may lead to rapid prioritization of processing of subsequent information (although the universality of the effect may be limited, see for example: [89, 90]). The upside of this plasticity of self is that people can quickly shift processing preferences to all sorts of information that have relevance for self. A potential downside is that associations with self can be easily created from the outside. Such associations may result in quick identification with information that propels neither personal nor societal development.

## Supporting information

S1 File Data. (XLSX)

S1 Text N2 following faces in experiment 2. The appendix contains an additional analysis of N2, which replicates the findings from experiment 1.
(PDF)
