# Supplementary material for: Prioritization of arbitrary faces associated to self: An EEG study
Source: PLoS One. 2018 Jan 2;13(1):e0190679. doi: 10.1371/journal.pone.0190679 (PMC5749812; doi:10.1371/journal.pone.0190679)
Supplement: S1 Text — The appendix contains an additional analysis of N2, which replicates the findings from experiment 1. (PDF) [file pone.0190679.s002.pdf]

S1 Text. The N2 evoked by the second stimulus in experiment 2 (face)

The idea behind the NM2 condition was to show the pure influence of the second stimulus on the subsequent ERPs. At the same time, the fact that it is a mismatching condition means that the second stimulus can be preceded only by a mismatching first one. This situation can introduce a potential bias. As seen in both reaction times and central-parietal P3 data, the identity of the first stimulus strongly influences processing of the second one. If the first stimulus is associated with self, then processing at the later stage is performed faster (as evidenced by faster RTs) and the central-parietal P3 is stronger, while the opposite pattern is true if the first stimulus is stranger-related. In NM2 trials the second stimulus is devoid of the facilitatory/inhibitory effect of the first stimulus with the same identity. For example: if the second stimulus is associated to the self, then in NM2 trials it will be preceded 50% of the time by friend- and 50% of the time by stranger-stimulus, but never by the self-related one. Therefore, if there is no difference caused by the second stimulus, we still can expect that reaction times will be slower, and the posterior P3 reduced, just because it is preceded by stimuli causing this effect.

In order to account for this, an additional analysis was performed on matching and mismatching trials combined together. The goal was to acquire unbiased means reflecting the influence of the first stimulus (STIMULUS-1) and the second stimulus (STIMULUS-2) on event-related potentials following the latter. In order to account for differences in the number of trials for each combination of stimuli we averaged averages across trials for all relevant combinations (see Supplemental Fig 1). For example, the estimates for self can be calculated as follows:

$$\mathbf{STIMULUS-1_{Self}} = \frac{|\mathrm{Label_{Self}}\rightarrow\mathrm{Face_{Self}}| + |\mathrm{Label_{Self}}\rightarrow\mathrm{Face_{Friend}}| + |\mathrm{Label_{Self}}\rightarrow\mathrm{Face_{Stranger}}|}{3}$$

$$\mathbf{STIMULUS-2_{Self}} = \frac{|\mathrm{Label_{Self}} \rightarrow \mathrm{Face_{Self}}| + |\mathrm{Label_{Friend}} \rightarrow \mathrm{Face_{Self}}| + |\mathrm{Label_{Stranger}} \rightarrow \mathrm{Face_{Self}}|}{3}$$

where $|\mathrm{Label_X} \rightarrow \mathrm{Face_Y}|$ stands for an average value of interest (the amplitude of ERP) in trials starting with a label X (first stimulus), followed by a face Y (second stimulus).

One-way ANOVA with repeated measures was performed on **STIMULUS**-1, and **STIMULUS**-2 trials separately, in order to estimate the influence of association of each stimulus (label and face) on the N2 following faces. The N2 was estimated based on the activity from electrodes AFz, AF3, and AF4, the same ones which were used for the N2 following faces in experiment 1. The time interval was slightly longer, and spanned between 200 and 350ms. It was chosen after inspection of the grand average across all conditions which showed that the onset of the N2 was earlier and the peak lasted longer than in experiment 1. The results showed that there was no influence of the first stimulus on the amplitude of the N2 ($F(2,34)=0.21$, $p=0.82$), but there was a strong influence of the second stimulus ($F(2,34)=4.25$, $p=0.023$, partial $\eta^2=0.20$). The pattern in **STIMULUS**-2 condition was the same as the one observed in experiment 1 after presentation of the face (figure 3), i.e. the N2 was reduced for self-associated face versus the others ($F(1,17)=13.3$, $p=0.002$), while there was no difference in its amplitude between friend and stranger-associated faces ($F(1,17)=0.3$, $p=0.61$).

These results provide converging evidence, obtained with independent sample and slightly changed experimental paradigm (changed order of the stimuli), that self-association of an unfamiliar face leads to subsequent prioritization of this face, as manifested by decreased amplitude of the N2. The same effect that has been observed when people perceive their real face [1-3]. This effect is not modulated by the identity of the stimulus which proceeds it in a sequence (a label in our task), and therefore reflects processes operating on a

timescale of the whole experiment rather than on the trial-by-trial basis. This interpretation converges with our interpretation of the N2 effect seen in experiment 1 as reflecting strengthened perceptual priors.

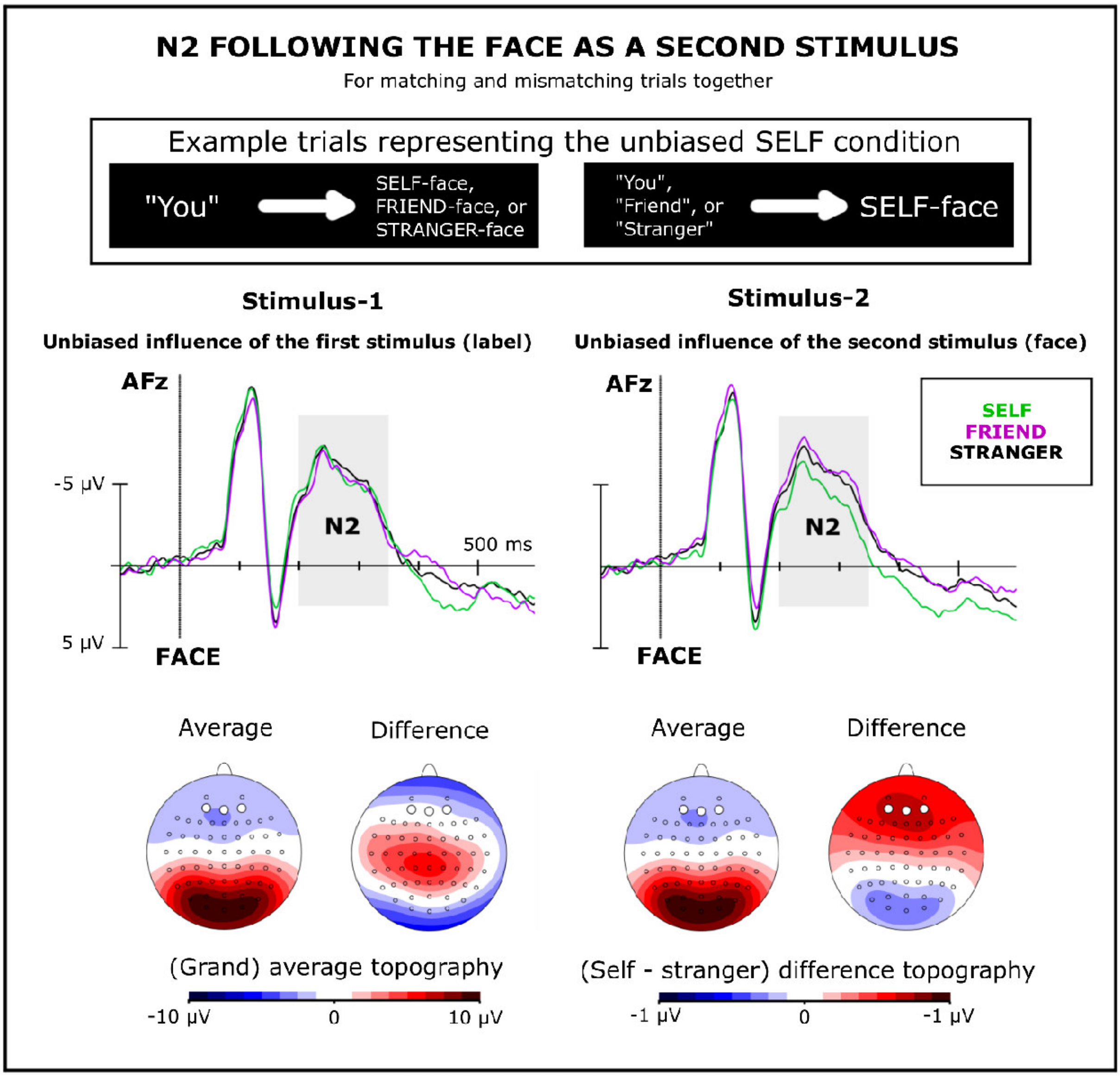

Supplemental Fig 1. The frontal N2 following faces in experiment 2. The influence of identity of the first (left side) and the second (right side) stimulus on the anterior N2 between 200 and 350ms after presentation of faces, for matching and mismatching pairs averaged together (see text for details). Pictures illustrate: (top) examples of trials, (middle) waveforms at the electrode AFz, and (bottom) the corresponding topographies of grand-average voltage and difference between self and others.

References

1. Sui J, Zhu Y, Han S. Self-face recognition in attended and unattended conditions: an event-related brain potential study. Neuroreport. 2006;17(4):423-7. doi: 10.1097/01.wnr.0000203357.65190.61. PubMed PMID: 16514370.
2. Keyes H, Brady N, Reilly RB, Foxe JJ. My face or yours? Event-related potential correlates of self-face processing. Brain Cogn. 2010;72(2):244-54. doi: 10.1016/j.bandc.2009.09.006. PubMed PMID: 19854553.
3. Scott LS, Luciana M, Wewerka S, Nelson CA. Electrophysiological correlates of facial self-recognition in adults and children. Cogn Brain Behav. 2005;(9):211-38.
